# Hemi-Cabrol Aortic Root Replacement in Complex Aortic Reconstructions

**DOI:** 10.1055/s-0038-1641606

**Published:** 2018-07-27

**Authors:** Ioannis Dimarakis, Isaac Kadir

**Affiliations:** 1 Department of Cardiothoracic Surgery, Wythenshawe Hospital, Manchester, United Kingdom

**Keywords:** aortic root, Cabrol procedure, complex aortic surgery, imaging in aortic disease, treatment outcome

## Abstract

The Cabrol aortic root replacement with subsequent modifications remains an extremely useful technique within the armamentarium of the aortic surgeon. The technical considerations detailed here allow for the uncompromised creation of a hemi-Cabrol anastomosis in complex aortic reconstructions.

Initially described by Cabrol et al in 1981, 1 the Cabrol aortic root replacement (CARR) has undergone several technical modifications as seen in relevant literature. Current expert consensus indicates that CARR should be used only when modified Bentall is not feasible, in view of the reported increased incidence of early- and long-term complications. 2

## Technique

### Patient A

**Fig. 1:**
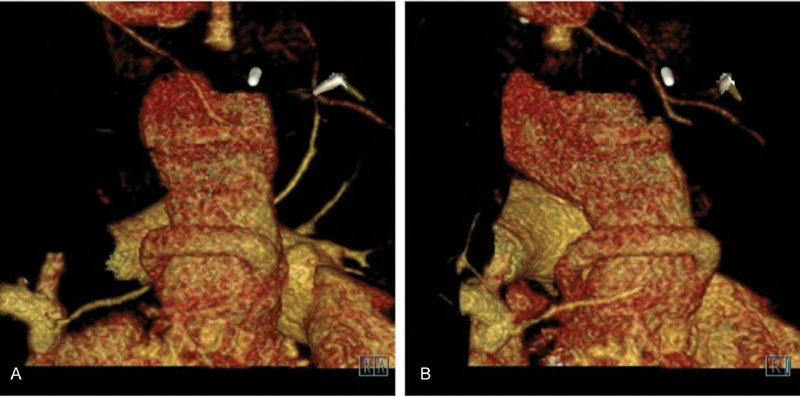
Computed tomography—three-dimensional reconstruction images: The 10 mm prosthetic graft is seen routed to the right side of the graft and anastomosed to the anterior surface of the tube graft ( **A** ). On a more lateral view the coronary graft may be seen passing anticlockwise into the left coronary artery ( **B** ).

A 63-year-old male patient had initially undergone a mechanical aortic valve replacement 17 years earlier for bicuspid aortic valve stenosis. He was referred with increasing shortness of breath on exertion and 5.6 cm dilatation of the ascending aorta on computed tomography. Aortic dimensions on intraoperative transesophageal echocardiogram measured at the level of the sinuses of Valsalva, the sinotubular junction, and the proximal ascending aorta were 5.2 cm, 5.2 cm, and 5 cm, respectively. During dissection of the aortic root, the left coronary button tissue was friable and therefore a hemi-Cabrol anastomosis with a 10 mm prosthetic graft was performed. This was routed to the right side of the tube graft and anastomosed to its anterior surface ( Fig. 1 ).

### Patient B

**Fig. 2:**
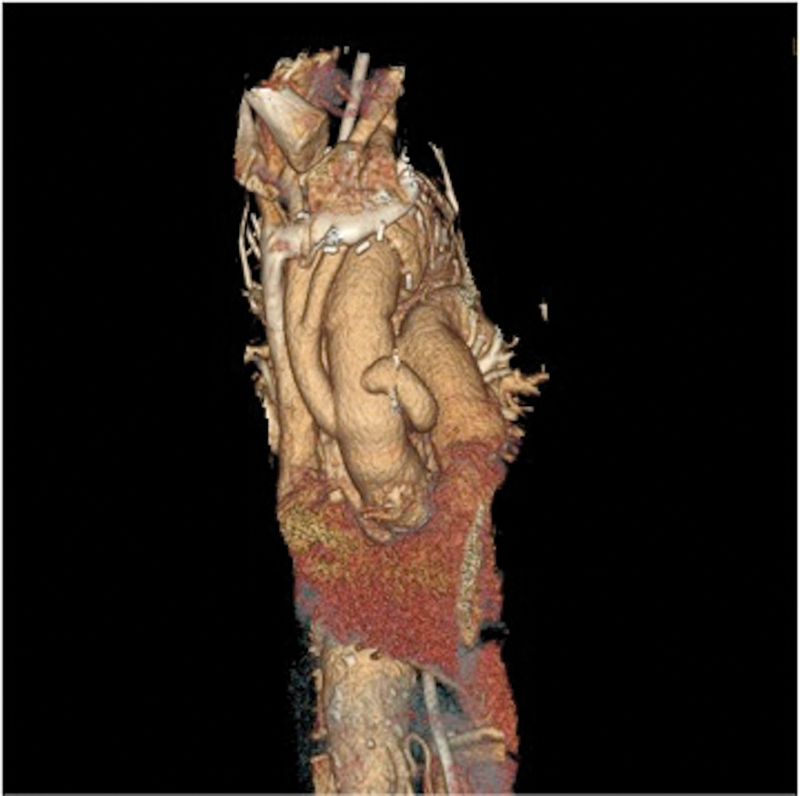
Computed tomography—three-dimensional reconstruction image: The 10 mm prosthetic graft on this occasion is routed to the left side of the tube graft due to the presence of the trifurcated graft.

A 67-year-old female patient was referred with an extensive aneurysm of the thoracic aorta, severe aortic regurgitation, and exertional angina. Embarking on a staged repair, via median sternotomy, we undertook biological aortic root, ascending aorta, and arch replacement with a conventional elephant trunk procedure. The head and neck vessel were reimplanted with a trifurcated graft. 3 In addition to being friable, the left coronary ostium had migrated due to the significant aneurysmal dilatation of the aortic root. On this occasion, a 10 mm prosthetic graft was routed to the left side of the tube graft as the presence of the trifurcated graft would compromise the function of the hemi-Cabrol ( Fig. 2 ).

## Comment

Cabrol aortic root replacement remains an invaluable technique especially in redo scenarios if coronary artery mobilization proves challenging. 4 In a similar fashion, inflammatory processes affecting the aortic root and coronary ostia may also require utilization of CARR. Finally, structural deterioration of stentless aortic valve implants may lead to calcification or severe adherence to the native aortic wall in which case reoperation may once again necessitate CARR. 5

In our experience, indications for CARR (beyond surgical mishaps during creation of coronary buttons) may include previous root surgery with dense fibrotic tissue or pseudoaneurysm formation in the region of the coronary ostium, aortic dissection with involvement of the coronary ostia, and pathological processes (i.e., endocarditis and inflammatory), leading to damage of the coronary ostia, previous stentless valve replacement, homografts, or Ross procedure requiring aortic root replacement.

We report two successful cases of hemi-CARR for different indications. Both patients had an uneventful procedure and in-hospital recovery. On follow-up, they remain well and postoperative imaging confirms conduit patency. Positioning of the left hemi-Cabrol graft may prove challenging in the face of complex aortic reconstructions because of the increasing likelihood of kinking and compression. Our description of routing the graft depending on the reconstructed anatomy avoids compression of the graft.
